# Supplementary material for: Bone marrow derived stromal cells from myelodysplastic syndromes are altered but not clonally mutated in vivo
Source: Nat Commun. 2021 Oct 25;12:6170. doi: 10.1038/s41467-021-26424-3 (PMC8546146; doi:10.1038/s41467-021-26424-3)
Supplement: Supplementary file 1 — Supplemental Information [file 41467_2021_26424_MOESM1_ESM.pdf]

Supplementary Information

Bone marrow derived stromal cells from Myelodysplastic Syndromes are altered but not clonally mutated in vivo

Johann-Christoph Jann[1], Maximilian Mossner[1], Vladimir Riabov[1], Eva Altrock[1], Nanni Schmitt[1], Johanna Flach[1], Qingyu Xu[1], Verena Nowak[1], Julia Obländer[1], Iris Palme[1], Nadine Weimer[1], Alexander Streuer[1], Ahmed Jawhar[2], Ali Darwich[2], Mohammad Jawhar[1], Georgia Metzgeroth[1], Florian Nolte[1], Wolf-Karsten Hofmann[1] and Daniel Nowak[1*]

[1]Department of Hematology and Oncology, Medical Faculty Mannheim of the Heidelberg University, Mannheim, Germany; [2]Department of Orthopedic Surgery, Medical Faculty Mannheim of the Heidelberg University, Mannheim, Germany

Supplemental Figure 1

Supplemental Methods

Supplemental Figure 1

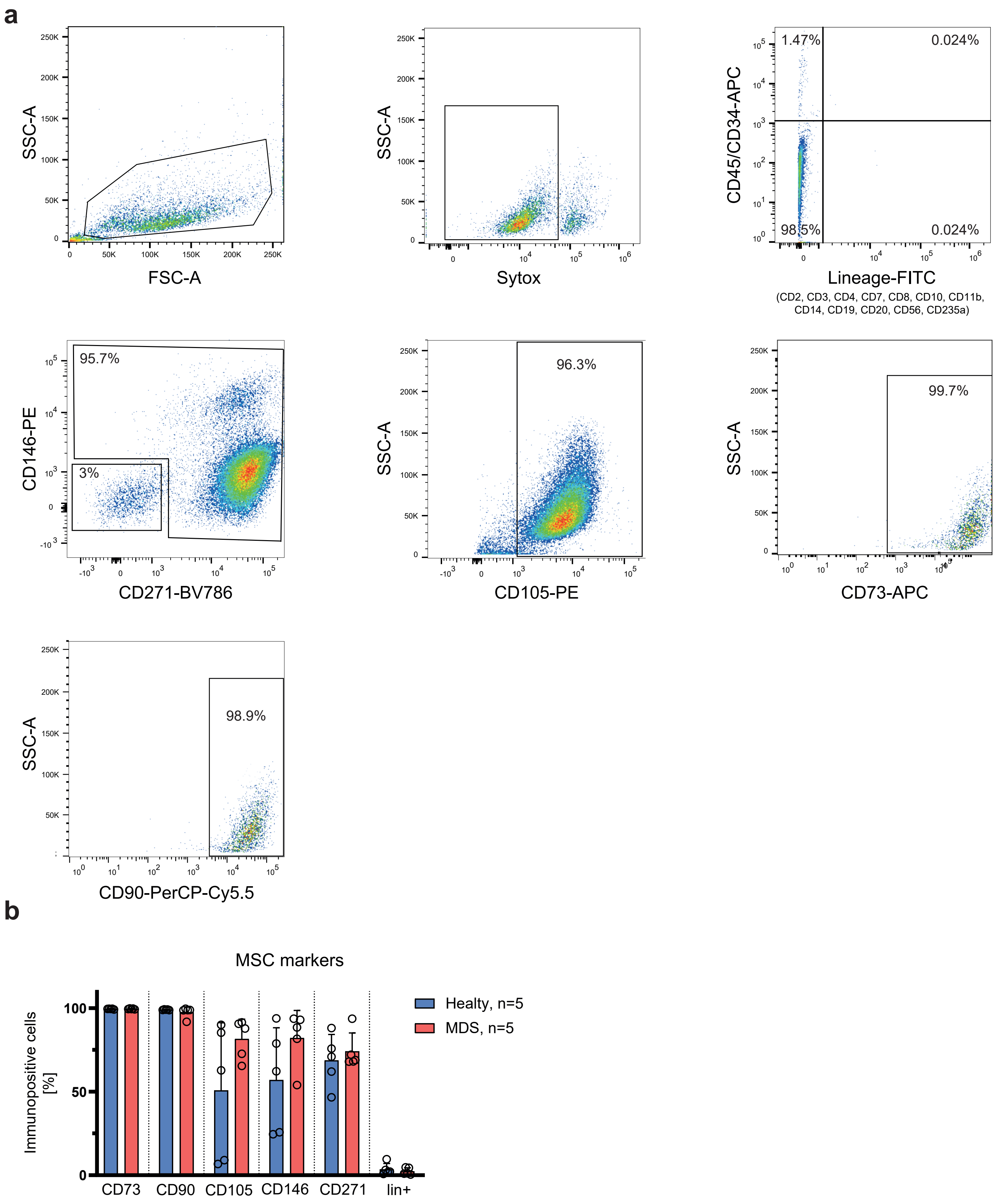

Legend:
(a) Representative FACS Plots of immunophenotypic characterization of in vitro cultured MSCs from a MDS patient.
(b) Summary of immunophenotypic characterization of MSCs from n=5 MDS and n=5 healthy with the above described FACS panel. Lin Lineage, bars show Mean + SD.

Supplemental Methods:

Whole exome sequencing

WES and data analysis was performed according to the following protocol: DNA from MSCs was isolated with QIAGEN ALT lysis buffer and bone marrow (BM) DNA using the All Prep kit (QIAGEN). 100-500ng of quality controlled high molecular weight genomic DNA was subjected to the Nextera DNA Flex Tagmentation kit (Illumina) followed by hybrid capture enrichment with the IDT xGene Research probe v1 set according to the manufactures' guidelines. The initial mutational calling strategy was performed as follows: fastq trimming by trimmomatic (v0.39), mapping with bwa mem (v0.7), MarkDuplicates (v2.20), realignment for batches of 5-10 samples with the gatk 3.8.0 framework, clipping of overlapping fw and rv reads with bamUtil ClipOverlap (v1.0.14) and recalibration with gatk 3.8.0 framework. Mutational calling was performed with Mutect2 (gatk v4.1.3) with an additional parameter of a panel of normal generated from n=100 BM MNCs from a variety of hematological neoplasms with identical library preparation. Only mutations with a PASS filter tag were further analyzed. To account for false positive calling due to LOH in the bone marrow sample we applied three strategies: First, we removed mutational calls in loci that called as copy number altered in the bone marrow using sequenza (v3.0.0). Second mutations with a MAF >5% were filtered and third we manually removed sites with clustered mutational calls in a distinct genomic region. Thereby 52/98 samples did not show any large scale LOH in the bone marrow and thus no genetic region was removed from calling. For the remaining minority of samples on average 9 Mb (1% of total hg19 length averaged for all samples) were removed from the calling region (**Supplemental Table 1**).

CFU Assay

Colony forming assays of MSCs were carried out by seeding 100-5000 either bone marrow mononuclear cells or pre expanded MSCs from P1 per 6 well and petri dish and cultured until colony formation with Medium XF (Miltenyi Biotec). Colonies were giemsa stained for better distinction and picked for DNA extraction (Qiagen ALT, DNeasy). Following that, genotyping was performed with surrounding primers and Sanger sequencing.

Telomer length assessment

20ng of genomic DNA was subjected to SYBRGreen multiplex qPCR according to Cawthon[1] using the following primers: telg FW: ACACTAAGGTTTGGGTTTGGGTTTGGGT-TTGGGTTAGTGT, telc RV, GTTAGGTATCCCTATCCCTATCCCTATCCCTATCCCTAACA, hbgu FW: CGGCGGCGGGCGGCGCGGGCTGGGCGGCTTCATCCACGTTCACCTTG, hbgd RV: GCCCGGCCCGCCGCGCCCGTCCCGCCGGAGGAGAAGTCTGCCGTT. Quantification was carried out using the ddCT method in triplicates.

γH2AX and RPA Quantification

MSCs from passage 1 were seeded on coverslips in 24-well plates and cultured for 5 days. Cells were fixed using 4% PFA for 10 min, RT and permeabilized using 0.15% Triton-X100 for 2 min, RT. After 1h block with 1%BSA, cells were labeled using anti-phospho-histone H2A.X (Ser139) antibody, clone JBW301 (EMD Millipore, Cat No: 05-636 1:1000) overnight at +4°C. Secondary Alexa Fluor 488 labeled goat anti-mouse antibody (ThermoFisher Scientific) were added for 2h. For RPA staining rabbit anti-RPA2 (pSer33) antibody (Novus Biologicals, NB100-

544, 1:1000) for overnight (+4°C) in blocking buffer and secondary Alexa Fluor 488 labeled goat anti-rabbit antibody (ThermoFisher Scientific) was used. Slides were mounted in Vectashield antifade mounting medium with DAPI (Vector Laboratories). The staining was evaluated using Axio Scope A1 microscope (Zeiss) equipped with a 63x objective. Samples were blinded and at least 50 cells in each sample were counted by an independent observer. The percentage of γH2A.X/ RPA positive cells as well as mean number of γH2A.X/ RPA foci per cell were calculated.

Senescence Assessment

For senescence associated β-Galactoidase (b-gal) activity assessment was carried out using flow cytometry, MSCs were thawed from passage 1, seeded in 12-well plates and cultured for 5 days, 37°C. Bafilomycin A1 (100 nM, Cell Signaling Technology) was added in fresh cell culture medium for 1h, 37°C followed by addition of 5-Dodecanoylaminofluorescein Di-β-D-Galactopyranoside (C_{12}FDG) in a final concentration of 33μM for 50 min, 37°C. Cells were washed in PBS, trypsinized and labeled using SYTOX Blue Dead Cell Stain (Thermo Fisher Scientific) followed by acquisition using a FACS Melody sorter/flow cytometer (BD Biosciences). For b-gal assessment using light microscopy, MSCs were seeded in 24-well plates and cultured for 5 days, 37°C. Cells were stained for b-gal activity using the Senescence β-Galactosidase Staining Kit (#9860, Cell Signaling Technology) according to the manufacturer's instructions. The images were obtained using an Axiovert 100 (Zeiss) and 5x objective.

RT-qPCR

MSCs from passage 1 were assessed for IL-6 expression using pre-designed IL-6 Taqman assay Hs00174131_m1 (Thermo Fisher Scientific). Data were normalized to the expression of beta-actin using the ddCt method in triplicates.

ELISA

MSCs' supernatants were collected at confluency from passage 1 and assessed for IL-6 production using Human IL-6 (DY206) DuoSet ELISA kits (R&D systems) according to the manufacturer's instructions.

Osteogenic differentiation assay

Osteogenic differentiation was initiated in confluent cultures of MSCs using α-MEM (Thermo Fisher Scientific, Schwerte, Germany) containing 10% FCS and 1% Pen/Strep. For osteogenic differentiation, the media were supplemented with 5 mM β-Glycerophosphat, 50 µg/ml ascorbic acid and 10 nM Dexamethason and changed all 2-3 days. Mineralized deposits were visualized by von Kossa staining after 21 days. Cells were fixed with iced 95% ethanol and 5% Isopropanol at 4°C for 1 hour and exposed to 25 mM silver nitrate under bright light for 10-20 min. Nodules stained with von Kossa stain after 21 days were quantified using ImageJ (Wayne Rasband, National Institutes of Health) according to Sens et al[2].

FACS analysis of MSC Markers

Cultured MSCs were analyzed by Flow cytometry of a BD FACS Melody device using the following antibodies: CD73-AD2, APC, Biolegend Cat No 344005, 1:20; CD90- 5 E10, PerCP-Cy5.5 BD Bioscience Cat No 328118, 1:100 ; CD105-266, PE, BD Bioscience Cat No 560839; 1:40; CD146- P1H12, PE Biolegend Cat No 361005 1:1000; CD271- C40-1457, BV786, eBioscience Cat No 743361, 1:20; lineage marker: CD45- HI30, APC, BD Bioscience, Cat No 555485 1:100; lin4-FITC BD Bioscience Cat No 562722, 1:100; CD34-561, APC, Biolegend Cat No 343607 1:20.

Supplemental References:

1. Cawthon RM. Telomere length measurement by a novel monochrome multiplex quantitative PCR method. Nucleic Acids Res. **37**(3):e21 (2009)
2. Sens C, Altrock E, Rau K, et al. An O-Glycosylation of Fibronectin Mediates Hepatic Osteodystrophy Through alpha4beta1 Integrin. J Bone Miner Res. **32**(1):70-81 (2017)
