# Supplementary material for: Bone marrow derived stromal cells from myelodysplastic syndromes are altered but not clonally mutated in vivo
Source: Nat Commun. 2021 Oct 25;12:6170. doi: 10.1038/s41467-021-26424-3 (PMC8546146; doi:10.1038/s41467-021-26424-3)
Supplement: Supplementary file 3 — Description of supplementary files [file 41467_2021_26424_MOESM3_ESM.docx]

**Description of Additional supplementary Files:**

File Name: Supplemental Data1.xlsx

Description: Primers used for targeted resequencing

File Name: Supplemental Data2.xlsx

Description: Overview of number of mutations with VAF >10% per sample and removed genomic region due to LOH in the bone marrow.

File Name: Supplemental Data3.xlsx

Description: Number and significance of mutated genes in the MDS cohort.

File Name: Supplemental Data4.xlsx

Description: Number and significance of mutated genes in the healthy cohort.

File Name: Supplemental Data5.xlsx

Description: Genes and samples tested with targeted resequencing.
