# Supplementary material for: Bone marrow derived stromal cells from myelodysplastic syndromes are altered but not clonally mutated in vivo
Source: Nat Commun. 2021 Oct 25;12:6170. doi: 10.1038/s41467-021-26424-3 (PMC8546146; doi:10.1038/s41467-021-26424-3)
Supplement: Supplementary file 9 — Reporting Summary [file 41467_2021_26424_MOESM9_ESM.pdf]

Corresponding author(s): Daniel Nowak
Last updated by author(s): Sep 20, 2021

Reporting Summary

Nature Research wishes to improve the reproducibility of the work that we publish. This form provides structure for consistency and transparency in reporting. For further information on Nature Research policies, see our Editorial Policies and the Editorial Policy Checklist.

Statistics

For all statistical analyses, confirm that the following items are present in the figure legend, table legend, main text, or Methods section.

n/a	Confirmed	
☐	☒	The exact sample size (*n*) for each experimental group/condition, given as a discrete number and unit of measurement
☐	☒	A statement on whether measurements were taken from distinct samples or whether the same sample was measured repeatedly
☐	☒	The statistical test(s) used AND whether they are one- or two-sided<br>*Only common tests should be described solely by name; describe more complex techniques in the Methods section.*
☒	☐	A description of all covariates tested
☒	☐	A description of any assumptions or corrections, such as tests of normality and adjustment for multiple comparisons
☐	☒	A full description of the statistical parameters including central tendency (e.g. means) or other basic estimates (e.g. regression coefficient) AND variation (e.g. standard deviation) or associated estimates of uncertainty (e.g. confidence intervals)
☐	☒	For null hypothesis testing, the test statistic (e.g. *F*, *t*, *r*) with confidence intervals, effect sizes, degrees of freedom and *P* value noted<br>*Give P values as exact values whenever suitable.*
☒	☐	For Bayesian analysis, information on the choice of priors and Markov chain Monte Carlo settings
☒	☐	For hierarchical and complex designs, identification of the appropriate level for tests and full reporting of outcomes
☒	☐	Estimates of effect sizes (e.g. Cohen's *d*, Pearson's *r*), indicating how they were calculated

Our web collection on statistics for biologists contains articles on many of the points above.

Software and code

Policy information about availability of computer code

Data collection

No special software was used for data collection.

Data analysis

Flowcytometry data was analyzed with the FlowJo software (version 10.5.3).
Standard statistical analyses as indicated in the figure legends were carried out with Graphpad Prism, Version 8.3.4
For analysis of next generation sequencing data we used the following publicly available software modules as outlined in detail in the methods and supplemental methods sections:
Trimmomatic v0.39;
bwa mem v0.7;
MarkDuplicates v2.20;
gatk v3.8.0;
bamUtil ClipOverlap v1.0.14;
Mutect2 (gatk v4.1.3);
sequenza v3.0.0;
MutSigCV v1.4;
vcf2maf (https://github.com/mskcc/vcf2maf);
R v3.6.3;
MutationalPattern v1.10;
deconstructSigs v1.9;
hisat2 v2.04;
cufflinks/cuffdiff v2.2.1;

For manuscripts utilizing custom algorithms or software that are central to the research but not yet described in published literature, software must be made available to editors and reviewers. We strongly encourage code deposition in a community repository (e.g. GitHub). See the Nature Research guidelines for submitting code & software for further information.

Data

Policy information about availability of data

All manuscripts must include a data availability statement. This statement should provide the following information, where applicable:

- Accession codes, unique identifiers, or web links for publicly available datasets
- A list of figures that have associated raw data
- A description of any restrictions on data availability

Raw sequencing data of exome sequencing and RNA sequencing underlying Figures 1B-H, 2A-B, 3I, J, M, N, 4A-J and 5B-J in this study is available for download EGA archive: https://ega-archive.org/ (accession ID EGAD00001006968 and EGAS00001000716). Availability of data is restricted to non-commercial research only use.

Field-specific reporting

Please select the one below that is the best fit for your research. If you are not sure, read the appropriate sections before making your selection.

☒ Life sciences ☐ Behavioural & social sciences ☐ Ecological, evolutionary & environmental sciences

For a reference copy of the document with all sections, see nature.com/documents/nr-reporting-summary-flat.pdf

Life sciences study design

All studies must disclose on these points even when the disclosure is negative.

Sample size

Sample size was n=98 MDS/myeloid neoplasia samples and n=28 samples from healthy donors as specified in Table 1. Samples size was determined on sample availability.

Data exclusions

There was no data exclusion.

Replication

To verify reproducibility of the explorative whole exome sequencing of bone marrow derived MSCs from MDS and myeloid neoplasia patients (Figure 1), individual patient samples were re-sequenced by alternative sequencing methods such as custom amplicon based targeted deep re-sequencing. Firstly, we performed custom amplicon based targeted deep re-sequencing (TDS) of a total of n=120 mutations in n=117 genes in n=12 samples (Supplemental table 4). Thereby, we confirmed a strong correlation of VAFs between exome sequencing and TDS (Figure 2A, r=0.85, p<0.0001). Notably, all mutations in ZFX and RANK were confirmed by TDS. Next, we exemplarily validated the strategy of exome sequencing MSCs versus BM MNCs as germline control compared to other potential germline specimen such as sorted CD3 positive T-cells from peripheral blood or buccal swab DNA from the same patient (Figure 2B). We observed a strong overlap for the called mutations with only 1 call (2%) being exclusive to the BM versus MSC comparison. Finally, to confirm the clonality of the detected mutations we genotyped CFU-Fs derived from pre-expanded MSCs from P1 in n=4 patient cases in a total of 23 colonies. This functional clonality assay likewise confirmed the previously detected mutations in corresponding ratios (Figure 2C, D). Therefore, all attepts at replication were successful.

Randomization

There was no rationale to randomize biosamples in this study because it was an explorative study to identify possible somatically acquired clonal mutations in primary MSCs of MDS patients. To analyse this question, randomization is not relevant because no treatment or functional test was applied, which would have made randomization into groups necessary. To exclude biases, there was no selection or exclusion of samples of any kind in this study.

Blinding

In order to obtain data in Figure 3A, B and C as well as Figure 3P and Q, where data acquisition was dependent on manual microscopy, samples were blinded to the examiners. In all other data sets, blinding was not necessary because as outlined above, there were no treatment or test-groups for blinded comparisons.

Reporting for specific materials, systems and methods

We require information from authors about some types of materials, experimental systems and methods used in many studies. Here, indicate whether each material, system or method listed is relevant to your study. If you are not sure if a list item applies to your research, read the appropriate section before selecting a response.

Materials & experimental systems

n/a	Involved in the study	
☐	☒	Antibodies
☒	☐	Eukaryotic cell lines
☒	☐	Palaeontology and archaeology
☒	☐	Animals and other organisms
☐	☒	Human research participants
☒	☐	Clinical data
☒	☐	Dual use research of concern

Methods

n/a	Involved in the study	
☒	☐	ChIP-seq
☐	☒	Flow cytometry
☒	☐	MRI-based neuroimaging

Antibodies

Antibodies used

anti-phospho-histone H2A.X (Ser139), clone JBW301 (EMD Millipore, Cat No: 05-636), dilution: 1:1000, host species: mouse, application: Fluorescence microscopy
anti-RPA2 (pSer33), polyclonal, (Novus Biologicals, Cat No: NB100-544), dilution 1:1000, host species: rabbit, application: Fluorescence microscopy
anti CD73-AD2, APC, clone AD2 , (Biolegend, Cat No: 344005), dilution 1:20, host species mouse, application: Flow cytometry
anti CD90-5 E10, PerCP-Cy5.5, clone 5E10, (BioLegend Cat No 328118), dilution 1:100, host species mouse, application: Flow cytometry
anti CD105-266, PE, clone 266 (BD Bioscience Cat No 560839), dilution: 1:40, host species mouse, application: Flow cytometry
anti CD146- P1H12, PE (Biolegend Cat No 361005) 1:1000, clone P1H12, host species mouse, application: Flow Cytometry
anti CD271-C40-1457, BV786, clone C40-1457, (BD BioScience Cat No 743361), dilution: 1:20, host species mouse, application: Flow cytometry
anti CD271-ME20.4, FITC, clone ME20.4 , (Biolegend, Cat No 345104), dilution: 1:20, host species mouse, application: Flow cytometry
anti CD45-HI30, FITC, clone HI30 (BD Bioscience, Cat No 555482), dilution: 1:100, host species mouse, application: Flow cytometry
anti CD45-HI30, PerCP-Cy 5.5, clone HI30 (BD Bioscience, , Cat No: 564106), dilution 1:100, host species mouse, application: Flow cytometry
anti lin4, FITC; BD Bioscience Cat No 562722), dilution: 1:100, host species mouse, application: Flow cytometry
anti CD34-561, FITC, clone 561 (Biolegend Cat No 343603), dilution: 1:20, host species mouse, application: Flow cytometry
anti CD235a-GA-R2, APC, clone GA-R2(HIR2) (BD Bioscience, Cat No 551336), dilution 1:100, host species mouse, application: Flow cytometry
anti CD31-WM59, APC.C7, clone WM59 , (Biolegend, Cat No 563652), dilution 1:1000, host species mouse, application: Flow cytometry

Validation

We have provided a link for the relevant data sheet for each antibody. The data sheet includes the manufacturer's validations statements, quality control procedures and relevant citations:
anti-phospho-histone H2A.X (Ser139); file:///C:/Users/dn2/AppData/Local/Temp/05-636_2476967.pdf
anti-RPA2 (pSer33); https://resources.rndsystems.com/pdfs/datasheets/haf008.pdf?_ga=2.65443054.1552278646.1631546511-1646686778.1631546511
anti CD73-AD2; https://www.biolegend.com/en-us/global-elements/pdf-popup/apc-anti-human-cd73-ecto-5-nucleotidase-antibody-6093?filename=APC%20anti-human%20CD73%20Ecto-5apos-nucleotidase%20Antibody.pdf&pdfgen=true
anti CD90-5 E10; https://www.biolegend.com/en-us/global-elements/pdf-popup/percp-cyanine5-5-anti-human-cd90-thy1-antibody-4515?filename=PerCPCyanine55%20anti-human%20CD90%20Thy1%20Antibody.pdf&pdfgen=true
anti CD105-266; https://www.bdbiosciences.com/content/bdb/paths/generate-tds-document.us.560839.pdf
anti CD146- P1H12; https://www.biolegend.com/en-us/global-elements/pdf-popup/pe-anti-human-cd146-antibody-9271?filename=PE%20anti-human%20CD146%20Antibody.pdf&pdfgen=true
anti CD271-C40-1457; https://www.bdbiosciences.com/content/bdb/paths/generate-tds-document.in.743361.pdf
anti CD271-ME20.4; https://www.biolegend.com/en-us/global-elements/pdf-popup/fitc-anti-human-cd271-ngfr-antibody-6209?filename=FITC%20anti-human%20CD271%20NGFR%20Antibody.pdf&pdfgen=true
anti CD45-HI30; https://www.bdbiosciences.com/content/bdb/paths/generate-tds-document.us.555482.pdf
anti CD45-HI30; https://www.bdbiosciences.com/content/bdb/paths/generate-tds-document.us.564106.pdf
anti lin4; https://www.bdbiosciences.com/content/bdb/paths/generate-tds-document.ca.562722.pdf
anti CD34-561; https://www.biolegend.com/en-us/global-elements/pdf-popup/fitc-anti-human-cd34-antibody-6035?filename=FITC%20anti-human%20CD34%20Antibody.pdf&pdfgen=true
anti CD235a-GA-R2; https://www.bdbiosciences.com/content/bdb/paths/generate-tds-document.us.551336.pdf
anti CD31-WM59: https://www.bdbiosciences.com/content/bdb/paths/generate-tds-document.us.563653.pdf

Human research participants

Policy information about studies involving human research participants

Population characteristics

Patient and healthy donor chracteristics are detailled in Table 1 of the manuscript.

Recruitment

The study cohort consisted of n=98 MDS and MDS associated myeloid neoplasia cases, who were treated at the Department of Hematology and Oncology of the Medical Faculty Mannheim, Heidelberg University, Germany (median age 73 years, range 44-86). As healthy controls, a cohort of n=28 healthy BM samples was acquired from patients undergoing hip replacement surgery (median age 75 years, range 36-84). These healthy donor samples all had normal blood counts, absence of active or prior malignancy and other confounding co-morbidity. All experiments were performed after written informed consent in accordance with the Declaration of Helsinki and approved by the Medical Ethics Committee of the Medical Faculty Mannheim. Detailed clinical characteristics of patients and healthy donors are provided in Table 1. There was no bias in selection of patient samples.

Ethics oversight

The use of primary human material for research purposes was evaluated and aproved by the Ethics committee of the Medical Faculty Mannheim of the Heidelberg University. The Ethics approval number is 2013-509N-MA.

Note that full information on the approval of the study protocol must also be provided in the manuscript.

Flow Cytometry

Plots

Confirm that:

☒ The axis labels state the marker and fluorochrome used (e.g. CD4-FITC).

☒ The axis scales are clearly visible. Include numbers along axes only for bottom left plot of group (a 'group' is an analysis of identical markers).

☒ All plots are contour plots with outliers or pseudocolor plots.

☒ A numerical value for number of cells or percentage (with statistics) is provided.

Methodology

Sample preparation	Bone marrow derived mesenchymal stroma cells (MSCs) were expanded adherently on plastic dishes by seeding 100µm-filtered full BM fragments and additionally seeding 5x10e6 mononuclear cells in StemMACS MSC Expansion Medium XF (Miltenyi Biotec) in T25 flaks (P0). After 2 days, the medium was changed and non-adhesive cells were removed. Cells were then further expanded with weekly medium changes. At 80% confluency this initial culture was trypsinized and split into 2-4 T75 flasks, corresponding to P1 with yields of approximately 60 000 cells per flask. Of note, the in vitro culture was carried out for the shortest possible period of time to obtain sufficient cell numbers for bulk DNA isolation. Cells were harvested before senescence or confluence. The median time of in vitro expansion before DNA preparation was 34 days, (95% confidence interval (CI) 22-50d).
Instrument	BD FACS Melody
Software	FlowJo (version 10.5.3)
Cell population abundance	The purity of the expanded MSCs was routinely confirmed to be completely depleted of residual hematopoietic cells (CD45 and lineage markers CD2, CD3, CD4, CD7, CD8, CD10, CD11b, CD14, CD19, CD20, CD56, CD235a), largely negative for endothelial CD31 and shown to be positive for stroma cell markers such as CD146, CD271, CD105, CD73, CD90 (Supplemental Figure 1).
Gating strategy	For sorting primary bone marrow derived MSCs for next generation sequencing, whole bone marrow mononuclear cells were initially gated over the 2D density of events on forward and side scatter (FCS/SSC) to exclude cell debris as well as hypergranulated cells and overly large cells. Subsequently, live cells were gated on absence of sytox blue staining. Live cells were then gated individually for negativity of the pan-leukocyte marker CD45. Live CD45- cells were then gated on negativity for the enddifferentiation marker of erythroid differentiation CD235a to exclude CD235a+/CD45- hematopoietic cells. CD235a-/CD45- cells were then sorted for positivity of the in vivo MSC marker CD271 and negativity of the endothelial marker CD31 as the most stringently sorted human bone marrow MSC fraction sequenced in Figures 5b-j. As further confirmation we also sorted the less stringent non-hematopoietic subfraction of CD45-, CD235a-, CD31+ and CD271+/- subfraction. The gating strategy has been outlined and depicted in the main figure 5a.

☒ Tick this box to confirm that a figure exemplifying the gating strategy is provided in the Supplementary Information.
